# Fragile X syndrome: a review of clinical and molecular diagnoses

**DOI:** 10.1186/s13052-017-0355-y

**Published:** 2017-04-19

**Authors:** Claudia Ciaccio, Laura Fontana, Donatella Milani, Silvia Tabano, Monica Miozzo, Susanna Esposito

**Affiliations:** 1 Pediatric Highly Intensive Care Unit, Department of Pathophysiology and Transplantation, University of Milan, Fondazione IRCCS Ca’ Granda Ospedale Maggiore Policlinico, Via Commenda 9, 20122 Milan, Italy; 2 Division of Pathology, Department of Pathophysiology and Transplantation, University of Milan, Fondazione IRCCS Ca’ Granda Ospedale Maggiore Policlinico, Milan, Italy; 3 Pediatric Clinic, Department of Surgical and Biomedical Sciences, Università degli Studi di Perugia, Piazza Lucio Severi 1, Loc. S. Andrea delle Fratte, 06132 Perugia, Italy

**Keywords:** Intellectual disability, Autism spectrum disorder, FMR1, Triplet expansion, Fragile X syndrome

## Abstract

**Background:**

Fragile X Syndrome (FXS) is the second cause of intellectual disability after Down syndrome and the most prevalent cause of intellectual disability in males, affecting 1:5000–7000 men and 1:4000–6000 women. It is caused by an alteration of the *FMR1* gene, which maps at the Xq27.3 band: more than 99% of individuals have a CGG expansion (>200 triplets) in the 5′ UTR of the gene, and *FMR1* mutations and duplication/deletion are responsible for the remaining (<1%) molecular diagnoses of FXS. The aim of this review was to gather the current clinical and molecular knowledge about FXS to provide clinicians with a tool to guide the initial assessment and follow-up of FXS and to offer to laboratory workers and researchers an update about the current diagnostic procedures.

**Discussion:**

FXS is a well-known condition; however, most of the studies thus far have focused on neuropsychiatric features. Unfortunately, some of the available studies have limitations, such as the paucity of patients enrolled or bias due to the collection of the data in a single-country population, which may be not representative of the average global FXS population. In recent years, insight into the adult presentation of the disease has progressively increased. Pharmacological treatment of FXS is essentially symptom based, but the growing understanding of the molecular and biological mechanisms of the disease are paving the way to targeted therapy, which may reverse the effects of FMRP deficiency and be a real cure for the disease itself, not just its symptoms.

**Conclusions:**

The clinical spectrum of FXS is wide, presenting not only as an isolated intellectual disability but as a multi-systemic condition, involving predominantly the central nervous system but potentially affecting any apparatus. Given the relative high frequency of the condition and its complex clinical management, FXS appears to have an important economic and social burden.

## Background

Fragile X Syndrome (FXS, OMIM #300624), also known as Martin-Bell Syndrome, was first described in 1943 by Martin and Bell as a form of intellectual disability (ID) following an X-linked inheritance pattern [1]. In 1969, Lubs first reported a distinct fragile site on the X chromosome that segregated with ID in 3 generations of a family, and in 1991, the association of the Xq27.3 fragile site with X-linked ID was confirmed [2, 3]. Therefore, FXS was defined as a clinical and cytogenetic entity and acquired its current name. Currently, it is known to be the second cause of ID after Down Syndrome (2.4% of all IDs), the first cause of inherited ID and the most prevalent cause of ID in males [4, 5]. The actual worldwide prevalence is estimated to range between 1:5000–7000 men and 1:4000–6000 women [5].

The diagnosis of FXS is based on the detection of an alteration of the Fragile X Mental Retardation-1 gene (FMR1), which maps at the Xq27.3 band [2]. More than 99% of individuals with FXS have an FMR1 loss-of-function caused by an increased number of CGG trinucleotide repeats in the 5′ untranslated (5′ UTR) region (typically >200 triplets). This allelic constitution is called a full mutation (FM) and produces the expression of the cytogenetic fragile site (FRAXA). Its result is a hypermethylated state of the FMR1 promoter, with consequent inhibition of FMR1 transcription and loss or heavy reduction of the protein product (FMRP). Therefore, the FXS phenotype is a direct consequence of the absence of FMRP; different types of FMR1 alterations (deletions encompassing the gene, intragenic deletions/duplications, single-nucleotide variants) are responsible for the remaining (<1%) molecular diagnoses of FXS [6].

The normal number of repeats within the FMR1 gene ranges from 5 to 44; a repeat number of 45–54 is considered to be a grey zone. A repeat number of 55–200 is called pre-mutation (PM), and it is associated with pathological conditions that differ from FXS : premature ovarian failure (POI) in females and fragile X - associated tremor/ataxia syndrome (FXTAS) in males (less frequently also in females) [6]. Given the phenotype breadth of the FMR variations, typical of trinucleotide expansion disease, we chose here to provide a review that is limited to the complete FXS phenotype, which affects individuals carrying a FM allele.

FXS inheritance does not follow a Mendelian pattern, but it depends on the number of trinucleotide repeats within the promoter of the FMR1 gene [6]: a transition from the PM to FM allele can occur because of the expansion phenomenon during the transmission of the maternal (very rarely of the paternal) X chromosome carrying a PM to her children [3, 4]. The frequency of individuals with the PM allele in the total population is approximately 1:850 for males and 1:257–300 for females [5, 7] - i.e., one in 300 females randomly chosen among the general population can potentially generate an affected male child.

Affected men have a typical phenotype, characterized by ID, long face, large and protruding ears, and macroorchidism [4, 6, 8]. Females heterozygous for the FM allele have a 30% chance of having a normal intelligence quotient and a 25% chance of having ID with an IQ < 70; nonetheless, they can present learning deficits and emotional difficulties [4]. The phenotype in females is strongly connected to the X inactivation (XCI) pattern.

However, FXS is much more than a simple ID; it is a multi-systemic condition that can potentially affect any apparatus because FMRP is widely expressed. The aim of this review was to gather the current clinical and molecular knowledge about FXS to provide clinicians with a tool to guide the initial assessment and follow-up of FXS and to offer laboratory workers and researchers an update regarding the current diagnostic procedures.

## Discussion

### General clinical features

**Fig. 1:**
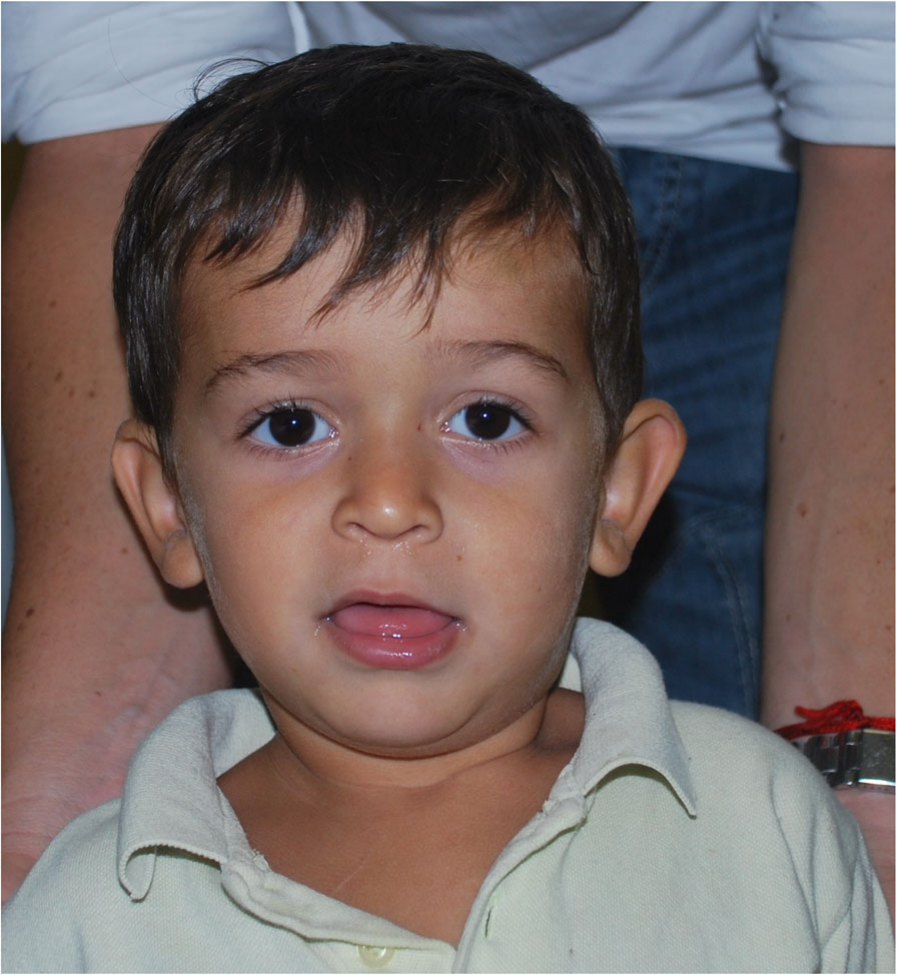
An FXS child showing long face, large and prominent ears, long palpebral fissures, broad philtrum, and facial hypotonia

Generally, prenatal and neonatal diagnoses are not possible with a negative family history because of the lack of ultrasound and clinical findings. At birth, the height, weight, and head circumference of FXS children are within the normal range [9]. The most prominent clinical features of the condition are summarized in Table 1. The height and weight seem to follow the normal growth curves; otherwise, the head circumference tends to reach the higher centiles: in prepubertal age, the majority of FXS children develop macrocephaly, with a head circumference larger than the 50th percentile [4, 6, 9]. The facial characteristics become more distinctive in early childhood, when the patients start showing a long narrow face and prominent ears [4, 9, 10]. This latter sign is one of the hallmarks of FXS, but it is often a relative parameter, as the narrowness of the faces of some affected males exaggerates their ear prominence [10]. Other reported facial features are the prominence of the jaw, a high-arched palate, puffiness around the eyes, long palpebral fissures, closely spaced eyes, epicanthal folds, strabismus, flat nasal bridge, broad nose, broad philtrum, and facial hypotonia (demonstrated by slackness of the lower jaw) [4, 6, 8–10] (Fig. 1). Notably, not all facial features are recognizable at a young age (most have been recorded only after puberty), and approximately 30% of young children with FXS will not have obvious dysmorphic features [6, 10].

**Table 1:** Clinical features of FXS males [4, 7, 9, 11, 13, 17, 18, 24, 26, 27, 32, 34, 36, 37, 51]

Features		Frequency
Face	Long/Narrow face	83%
	Macrocephaly	81%
	Prominent ears	72–78%
	High-arched palate	94%
	Prominent jaw	80%
	Facial hypotonia	NA
	Eye puffiness	NA
	Closely spaced eyes	NA
	Long palpebral fissures	NA
	Epicanthal folds	NA
	Flat nasal bridge	NA
	Broad nose	NA
	Broad philtrum	NA
Central nervous system	EEG anomalies	74%
	Epilepsy	10–20%
	Brain MRI anomalies	Up to 50% of patients with neurologic comorbidities
Neuropsychiatric involvement	Psychomotor delay	~100%
	ID	~100%
	Aggressiveness	90%
	Attention problems	74–84%
	Hyperactivity	50–66%
	Anxiety Disorder	58–86%
	ASD	30–50%
	Sleep problems	30%
	ADHD	12–23%
	Depression	8–12%
Musculoskeletal system	Joint hypermobility	50%
	Pectus excavatum	50%
	Flat feet	29–69%
	Spine deformity	6–9%
Cardiovascular	Mitral valve anomalies	3–12%
	Aortic root dilatation	25%
Eye	Refractive errors	17–59%
	Strabismus	8–40%
	Nystagmus	5–13%
Other	Macroorchidism	63–95%
	Obesity/overweight	53–61%
	Recurrent otitis media	47–97%
	Gastrointestinal complaints	31%
	Soft skin	NA

*Abbreviations*: *NA* not available, *ID* intellectual disability, *ASD* autism spectrum disorder, *ADHD* anxiety disorder/hyperactivity disorder

The most important clinical abnormality associated with defects of the FMR1 gene is global developmental delay/ID. The psychomotor delay involves both walking age (mean = 2,12 years) and age at first words (mean = 2,43 years) [11]. Both males and females with FXS present a wide range of learning disabilities in the context of normal, borderline IQ or mild to severe ID [12]. The IQ of males with FM varies with studies, with a mean value of 40–51 [11–13]; 68% of FM males have an IQ score lower than 50, while 18% have a score above 70 [11]. The IQ score directly correlates with the level of FMRP production: higher levels of FMRP are found in individuals with an IQ above 70, showing only moderate emotional and learning difficulties [4, 14, 15]. Similarly, those individuals with “size-mosaicism” (full mutation plus premutation, grey zone or normal alleles) have higher IQs than those without mosaicism [15]. Females with FM present a wider range of phenotypic characteristics than men, depending on the XCI pattern: 70% of FM women present with some degree of cognitive impairment [4].

### Neurological features

An important comorbidity in FXS is epilepsy. Reports have suggested a prevalence of seizures among FXS children, present in 10–20% in boys and 5–10% in girls [13, 16, 17]. Complex partial seizures have been reported to be the most frequent among FXS patients with epilepsy (89.3%) [13, 16], followed by generalized tonic-clonic seizures (46.4%), and simple partial seizures (25%). The latter type is always associated with another type of epilepsy; febrile convulsions have been reported in 7.1% of patients with epileptic seizures [13]. The age of onset is usually between 2 and 10 years, and this comorbidity typically disappears with growth, although 25% of FXS patients continue to have epilepsy into their adult years [13, 16]. Seizures have usually a low frequency of recurrence and sometimes manifest themselves following intercurrent infections or exposure to other environmental factors [13]. Epilepsy usually has a good response to therapy [13, 16]. Most patients control their seizures with antiepileptic drugs (AEDs); only 7% of the patients need more than one drug, and 10% of the patients do not need any therapy [13].

Independently from epilepsy, patients with FXS also have a higher prevalence of EEG abnormalities (74%) [13, 18]; these abnormal EEG findings, however, may not always manifest with seizures and/or a subsequent diagnosis of epilepsy. In a study by Hear et al., 47% of FXS patients exhibited slowing of the posterior dominant rhythm for age, and 42% had focal spikes from various anatomic regions [18]. Nevertheless, consistent with seizure remission with age, 35% of children showed normalization of the EEG background after the age of 8 years, and when present, they are more often nonspecific and limited to only one location [13, 18].

MRI is usually normal [13, 18]. When anomalies are found, these are more frequently diffuse atrophy and cortical thickness, increased whole hemispheric and lobar cortical volume, and increased cortical complexity [13, 19]. These aspects are consistent with the decreased pruning and increased spine density and length and with the presence of an immature spine, as reported in FXS patients and mice [19–21].

Further reported findings are atrophy of the cerebellar vermis, thinning of the corpus callosum, hippocampal anomalies, enlarged fourth ventricle, lacunar infarction of the basal ganglia, and mesial temporal sclerosis; the latter describes only cases of refractory seizures due to recurrent prolonged episodes of status epilepticus [13, 18]. A recent study by Hall et al. discovered increased fractional anisotropy in patients with FXS in the left and right inferior longitudinal fasciculus, right uncinate fasciculus and left cingulum hippocampus compared with that in controls; additionally, this aspect could be attributed to the aberrant pruning and axon growth dysregulation, resulting from FMRP reduction [22]. All of these MRI anomalies in brain morphology correlate negatively with cognitive performance in FXS children [19].

### Neuropsychiatric features

Over the years, FXS has been associated with several neuropsychiatric and neuropsychological phenotypes, showing that ID is rarely presented alone in this disease. Psychomotor delay is the first sign of an upcoming ID in the scholar age, and it is quite an early finding. The developmental profile of infants with FXS deviate from that of the general population by 6 months of age, involving all domains of development (fine motor, visual reception, expressive communication, and receptive communication) [23]. The average functional level of male patients shows an improving trend until the age of 25, even remaining below that of the general population. Then, patients enter a relatively stable phase until the age of 50; at that time, the skills of FXS males begin to worsen [24].

FXS patients are considered to be at a high risk of developing one or more neuropsychiatric disorders. An association between autism and FXS was first noted in the early 1980s, and a growing number of reports of further neuropsychiatric conditions emerged in the following years. Among those, the associations FXS/autism and FXS/Anxiety Disorder Hyperactivity Disorder (ADHD) are the most studied [11, 17, 25–33].

The autism-like presentation of many FXS males is known since the earliest study on the psychological characterization of the syndrome [25]. It is estimated that 30–50% males and 25% females with FXS have an Autism Spectrum Disorder (ASD) as a comorbidity [26, 27, 30]. Some studies suggested that there is an age-related improvement in some but not all ASD symptoms across adulthood for FXS men [27, 28]: autism is diagnosed in approximately 49% of children but 41% of adolescents/adults [27]. Nevertheless, the ASD-related impairment seems to be less severe in FXS individuals than in those with non-syndromic ASD [29]. In recent years, the change in the diagnostic criteria for ASD, due to the transition from DSM-IV TR to DSM-5 in 2013, is modifying this rate of incidence because the prevalence of ASD diagnoses is lower using the DSM-5 criteria across all age and sex groups [28, 30]. Only 50% of males diagnosed with ASD using the DSM-IV-TR parameter still fulfil the ASD criteria using the DSM-5. This gap is even broader for females; only 30% of FXS girls meet an ASD diagnosis changing from DSM-IV-TR to DSM-5 [30]. Anyway, given the high prevalence of autistic features in FXS and the fact that sometimes it represents the only sign of the syndrome, all children affected by ASD, especially boys, should be tested for FMR1 [6].

ADHD is considered one of the most common comorbidities in FXS, with more than one-half of male patients fulfilling the diagnostic criteria at some point in their lives. The prevalence of ADHD spectrum symptoms is 54–59%, a higher rate than that in individuals with isolated ID or different neuropsychiatric disorders [31]. The complete diagnostic criteria of ADHD are fulfilled by 12–23% of the FXS subjects [11, 32]. Preschool boys seem not to differ from typically developing controls in the mean level of ADHD symptoms and reach their peak at school age (5–6 years) [32].

As part of the ADHD spectrum, isolated hyperactivity also has a high incidence, with 50–66% of FXS children being affected [11, 17, 27, 33]; attention problems are well represented too, with an overall prevalence of 74–84% [17, 27]. Anxiety disorder is another frequent trait of FXS subjects, with a prevalence that largely varies with studies in the range of 58–86% [17, 27, 34]. Cordeiro et al. demonstrated that in a group of 58 males and 39 females with FXS aged 5–33 years, 86.2% of males and 76.9% of females met the criteria for at least one anxiety disorder [34]. Both anxiety and attention problems seem to follow an increasing trend with age [27].

The same trend also characterizes depression, which is prevalent in adolescents/adults rather than in children and has an overall prevalence of 8–12% in FXS individuals [17, 27]. This may not always manifest, but it can be revealed by withdrawal or increased aggression rather than by sadness, anhedonia, or irritability [26].

Other common neuropsychiatric conditions are the following: pervasive developmental disorder [11, 23], stereotypies (mostly hand/finger mannerisms) [11, 35], sleep problems [36], specific or social phobias [34], selective mutism [34], restricted interests [35], compulsive and ritualistic/sameness behaviours [35], self-injurious behaviour [35], and aggressiveness [27]. This latter aspect has been fully examined recently by Wheeler et al., who demonstrate that 90% of individuals with FXS, both males and females, were reported to be engaged in at least one aggressive act in the previous 12 months [37]. Sleep disorders affect approximately 30% of FXS children of both sexes and manifest themselves as difficulties falling asleep, frequent night-time awakenings, and early awakening in the morning [36].

It is then clear that aside from ID, the neuropsychiatric/neuropsychological profile of FXS is complex, and it has been demonstrated that overall, when using DSM-IV-TR criteria, 73% of FXS patients can be diagnosed with at least one axis I psychiatric disorder [11]. This incidence can indeed be underestimated because the assessment of psychiatric symptoms in patients with FXS is often complicated by limitations in the accuracy of self-reporting and insight, atypical manifestation of some symptoms in the context of ID, and the relative lack of validated assessment tools. For example, limited expressive language and social reciprocity impairments often prevent a reliable communication of symptoms. Diagnostic overshadowing occurs when psychiatric symptoms are not appreciated as a co-morbid problem in a patient with ID but are attributed only to the disability itself [26].

### A real syndrome: multi-systemic involvement

The neurologic/neuropsychiatric presentation certainly is the hallmark of the syndrome, but FXS also shows an association of various medical problems that may or may not be present; however, when manifested, it can worsen the phenotype and complicate the clinical management of these patients.

Since the first reports of FXS, it has been clear that the condition shares some features with the connective tissue disorder (CTD) spectrum. Although a specific abnormality of the connective tissue has not yet been shown, the prevalence of connective tissue signs has suggested that there is an underlying connective tissue anomaly, perhaps similar to that observed in CTDs (in particular, Marfan Syndrome and Ehlers-Danlos Syndrome); the precise association between FXS and signs of connective tissue abnormality still awaits biochemical and molecular explanation [38]. The skin can be soft [24, 39, 40], and joint hypermobility is present in about half of the patients, affecting predominantly the small joints (mostly metacarpal-phalangeal joints) [6, 24, 39, 40]. Skeletal signs may include a high-arched palate, scoliosis, pectus excavatum, and flat feet [6, 9, 24, 39, 40].

Connective tissue fragility also involves the heart because FXS patients can develop cardiac defects similar to those observed in CTDs. Recurrent findings are aortic root dilatation (approximately 25% of the patients) and mitral valve prolapse (3–50%) [9, 38–40]; this latter feature is frequent in the general population but has a higher prevalence in CTDs. Hyperarousal (i.e., faster heart rate) and reduced parasympathetic vagal tone have also been documented [41]. In adult age (>40 years), FXS patients tend to develop the common cardiovascular problems shared by the age-matched general population, such as hypertension (24.2%) and heart rhythm disorders (24.2%) [42].

The function of the gastrointestinal system in individuals with FXS has not been well studied yet. Given the presence of connective tissue signs, hypotonia and connective tissue anomalies could contribute to some of the gastrointestinal problems reported in this condition, such as gastro-oesophageal reflux, constipation, and loose bowel movements. In a study by Utari et al. including FXS males and females aged 40–71 years, a prevalence of gastrointestinal problems of 30.6% was reported [42]. Nonetheless, the literature lacks studies documenting an effective increased incidence of gastrointestinal involvement compared with that in the general population.

The genitourinary system seems to be affected only in males, where pubertal macroorchidism is considered a hallmark of the condition, shared by 80-95% of adults, but it is less common in prepubertal boys [4, 6, 8, 9, 39, 40]. In adult FXS men, the mean testicular volume is approximately 50 mL (normal mean testicular volume: <25 mL) [8]. Given the presence of ID, most FXS men do not have any real fertility complaints, even if 1% of them have been reported to reproduce [4].

Ocular anomalies are known since the first reports of the condition and affect at least 25% of FXS children and a greater number of FXS adults [43, 44]. Strabismus and refractive errors have a higher prevalence in FXS than in the general population [6, 8, 24, 45]: that prevalence, considerably variable with studies, is 8–40% for strabismus and 17–59% for refractive errors (primarily hyperopia and astigmatisms, but myopia is also reported) [8, 24, 43, 44, 46]. Nystagmus has been identified as a less rare finding (5–13%) [43, 46]; other observable ocular features include palpebral ptosis and convergence insufficiency [46].

FXS children tend to have recurrent otitis media, which may lead to conductive hearing loss [6, 8, 9, 11]. These patients already have poor expressive language skills, so it becomes fundamental that any possible otologic problem is promptly treated to avoid interference with speech improvement [47]. It has been reported that language skills are better among children who did not have recurrence of this complication [48].

Metabolic problems are common and well reported, with obesity and overweight being quite frequent in both sexes [6, 8, 42]. Studies conducted in FXS adults reported an incidence of 53–61% for obesity/overweight [42, 49]. Males are more frequently overweight, while women tend to reach obesity [42]. Furthermore, in males with FXS, the serum levels of HDL are shifted to lower numbers across all age ranges, but conversely, their triacylglycerol levels are higher than those of the general population [49, 50].

### Differential diagnosis

The differential diagnosis of FXS includes syndromic forms of ID but also non-syndromic psychomotor delays/ID. The differential diagnosis includes Sotos Syndrome, Prader-Willi Syndrome, Klinefelter Syndrome, and FRAXE [6, 51, 52]. These conditions share the following phenotypic features with FXS:

- Sotos Syndrome: ID, macrocephaly, behavioural problems, and epilepsy [6, 51, 53]
- Prader-Willi Syndrome: developmental delay, elements of facial appearance, sucking problems in neonatal age, obesity, and genital anomalies [6, 51, 54]
- Klinefelter Syndrome: ID (20%) and genital anomalies [51]
- FRAXE: ID (generally milder than FXS), language impairment, hyperactivity, and autistic behaviour [6, 51].

Angelman and Rett Syndromes may also be considered for the differential diagnosis, even if their typical presentation differs from that of FXS children. Shared features are ID, language impairment, and autistic behaviour. An Array-CGH can be performed to exclude cytogenetic rearrangements responsible for ID. When genetic testing is not helpful, isolated ID, autism, or ADHD must be considered [52].

### The FMR1 gene and FMRP: from triplet expansion to pathology

FXS is associated with a rare fragile and unsteady site on Xq27. This site, named FRAXA, was originally observed as a non-staining gap, break or constriction in the metaphase chromosomes placed under selective culture conditions, such as folic acid or thymidine deprivation [2, 55, 56]. In 1991, an association between FXS and alterations of the FMR1 gene was identified, located at the FRAXA locus [3]. The FMR1 gene product, FMRP, is involved in the regulation of post-transcriptional RNA metabolism, playing an important role in synaptic plasticity, dendrite and axon development, and underlying learning and memory. FMRP acts as an organizer of both mRNA transport (shuttle protein), targets mRNA translation (RNA-binding protein) and is involved in a feedback loop by controlling its own local protein levels [57].

The absence of FMRP derives, in most cases, from a dynamic mutation consisting of variable expansion of a trinucleotide (CGG) repeat in the 5′ UTR of the FMR1 gene. The size of the CGG repeat in normal individuals ranges between 5 and 44, and it is usually stably transmitted throughout generations. Alleles with 45–54 repeats are defined as intermediate, borderline or “grey-zone” (GZ). Carriers of GZ alleles do not show an FXS phenotype but can present with peripheral neuropathy, ataxia, anxiety and/or depression, and clinical symptoms similar to those of Parkinson patients, including bradykinesia, rigidity, memory complaints, and a positive response to dopaminergic medications [58]. PM carriers have a number of repeats that ranges from 55 to 200. PM disorders were first identified in 1991 with the discovery of an increased incidence of early menopause (prior to the age of 40 years) in female carriers [59]. Twenty percent of PM females manifest an FMR1-related POI [6], while FXTAS occurs in PM males (rarely in females) and is characterized by late-onset, progressive cerebellar ataxia and intention tremor [6, 60]. The necessary findings to confirm an FXTAS diagnosis are an FMR1 PM associated with an MRI showing white matter lesions in the middle cerebellar peduncles and/or brain stem (the major neuroradiologic sign) and intention tremor or gait ataxia (the two major clinical signs) [6]. The prevalence of FXTAS is estimated to be 40–45% overall for males and 16% for females with PM older than 50 years [60, 61]. The incomplete penetrance of POI and FXTAS phenotypes makes it difficult to predict whether a carrier would develop one of the PM-associated conditions. A major role in determining this penetrance is certainly due to environmental and genetic/epigenetic factors that may influence the susceptibility to phenotype expression. These latter include CGG repeat length, FMR1 mRNA concentration, XCI, translation of the repeat sequence, and any genomic asset able to influence FMR1 expression [62]. In recent years, it has emerged that a low-normal repeat number may also have important clinical implications: a CGG number below 26 can be considered a risk factor for cognition disability and mental health problems [63]. Therefore, there is growing awareness that there is a fine homeostatic equilibrium of FMRP expression levels, so that both high and low numbers of CGG repeats could alter brain function and alleles previously considered benign (<26 or 45–54) may lead to neuropsychiatric manifestations; anyway those are preliminary data that still need to be confirmed by additional studies. In FXS patients, the CGG trait is expanded (FM), with a dimension greater than 200 repeats. This expansion results in transcription silencing and the consequent absence of FMRP, due to hypermethylation of the CpG islands adjacent to the expanded trinucleotide repeats and heterochromatin conformation of the FMR1 promoter region. Conversely, GZ and PM alleles are unmethylated.

The risk of a PM allele becoming an FM allele is correlated with the number of CGG trinucleotide repeats, with nearly all alleles with ≥100 repeats expanding to FM in the next generation when transmitted by the mother; when the PM is carried by the father, small increases in the trinucleotide repeats may occur in meiosis but typically do not result in FM [6, 64, 65]. It has been estimated that 66% of maternal and paternal PM alleles change by one or more repeats in the offspring, even though with a significant difference in number of repeat expansions among maternal and paternal transmission: for alleles with <70 repeats, paternal alleles have a greater likelihood for instability; however, for larger alleles, maternal transmissions are more often unstable [64]. Approximately 17% of intermediate alleles are unstable, and maternal GZ alleles may evolve in PM (but not in FM) in future generations [6, 65]. Predictably, GZ alleles ranging from 50 to 54 repeats are less stable than those with <50 repeats and can more often evolve into PM ones [65]. Regarding normal-range alleles, 0.2% of maternal and 1.5% of paternal alleles exhibit an increasing size upon transmission [65].

Beyond CGG repeat size, one of the major factors influencing FMR1 stability is the presence of AGG triplets interspersed within the FMR1 repeated region. One or two AGG interruptions are usually interposed with CGG repeats in FMR1 (most commonly on the 5′ end of the repeat tract), and this occurs in 94% of the general population alleles [66]. Conversely, FXS alleles containing long stretches of uninterrupted CGG triplets at the 3′ end usually do not show any AGG at the 5′ end [66]. Maternal alleles with no AGGs have the greatest risk for FM expansion, while the presence of even a single AGG significantly reduces this risk, especially for alleles with <70 repeats. When the repeat number exceeds 70 triplets, the allele shows high instability even when AGG interruptions are present, and when the CGGs expand beyond 90, AGG interruptions do not have any ability to block the triplet growth [64].

A decrease in CGG repeat number through generations, although rare, is also possible: retractions from FM to PM and from PM to normal size have both been widely reported, while retraction from FM to normal size appears to be sporadic [67–69]. In a study by Nolin et al., by screening 1040 FMR1 pedigrees, repeat contraction had been observed in 2.3% of maternal and 5.7% of paternal transmissions [64]; previous work of the same research group had already shown a similar rate of contraction for maternal PM alleles (3,1%) [65]. Retraction occurs post zygotically because of an excision of a variable number of trinucleotides, resulting in a mosaic normal size/GZ/PM/FM [68, 69]. Generally, mosaics have a higher percentage of the larger allele [69].

Mosaicism is a source of phenotypic variability, and it can be observed in both sexes, with a higher incidence in males. The prevalence of mosaicism in FXS males largely varies among studies, in the range of 12–41% [6, 67]. Both repeat-size mosaicism (e.g., FM/PM, FM/GZ) and methylation mosaicism have been described; in the latter, FM has varying degrees of methylation from tissue to tissue [6, 68–70]. Somatic FM/deletion mosaicism has also been reported because deletion can occur mitotically during embryonic cell divisions, usually before the 11th week, with the result of two distinct subpopulations of cells carrying the deletion and FM alleles [71].

In addition to the FMR1 promoter expansion, a small number of FXS cases (<1%) are caused by mutation in the coding region or deletion of the FMR1 gene [6]. Mutations account for approximately 4% of FXS patients meeting the clinical criteria for FXS but with a normal range of CGG repeats [72]. Point, missense, nonsense, frameshift, and UTR region mutations have all been described [72–75]. Some of the variants lately identified still require investigation to definitively classify them as pathological because the FMRP is sometimes still present to some degree in carriers [73]. In line with this genotype-phenotype correlation, a recent study by Teckan et al. showed that approximately 30–50% of all FMR1 missense SNPs could be associated with diseases using an in silico approach [75].

Another important issue to consider, when approaching a diagnosis in females, is X chromosome inactivation (XCI), consisting of the silencing of one of the two X chromosomes in mammalian females. Normally, XCI occurs randomly; however, under particular conditions, preferential XCI can be observed. In the case of FMR1 mutation carriers, if the mutated chromosome is preferentially inactivated, FMRP is produced by the normal allele, and the resulting phenotype would be less severe [76].

### Diagnostic procedures

Initially, the diagnosis of FXS was based on the cytogenetic evaluation of the presence of FRAXA in peripheral blood lymphocytes (PBLs). However, this procedure was time consuming and difficult to interpret, required specific technical skills and was also unable to distinguish between FRAXA and the other neighbouring fragile sites on Xq [55]. To overcome some of these limitations and improve the detection rate of FRAXA, fluorescence in situ hybridization (FISH) with DNA probes was then introduced [77].

Cytogenetic analyses were replaced by Southern blot analysis of DNA from peripheral blood after digestion with specific restriction endonucleases [78], and finally by PCR. Southern blot analysis can detect all FMR1 alleles, including normal, PM, and FM, and can determine the methylation status of the FMR1 promoter region; however, it is time consuming, relatively expensive, and similarly to the previously used approaches, difficult to interpret. Standard PCR plus Southern blot analysis has been considered the gold standard for FMR1 molecular diagnosis for a long time, even if it provides a low-resolution estimation of the repeat number [66, 79, 80]. Standard PCR, based on the direct amplification of the CGG-repeat using flanking primers, is faster and highly sensitive to detect FMR1 repeats in the normal and PM range; however, it could only reveal alleles with up to ~300 repeats in males and up to ~160 repeats in females and it therefore fails to identify the large CGG expansions (e.g. more than 300 CGGs) [81].

The limitations of the PCR plus southern Blot technique lead to the development of new PCR-based procedures able to detect all FMR1 alleles. Triplet primed PCR (TP-PCR) was designed: it is a procedure in which the forward PCR primer is located upstream the CGG region and the other overlaps the CGG repeat and the adjacent unique sequence; after PCR cycles, the CGG repeat number can be determined by fragment sizing of PCR amplicons using capillary electrophoresis [66, 82]. TP-PCR is the evolution of previous PCR protocols; this procedure allows the simultaneous amplification of both the full-length FMR1 alleles (using PCR primers flanking the repeated region) and CGG triplets (using a third primer, complementary to the FMR1 triplet repeat region) in the same PCR reaction. TP-PCR-based procedures became the gold standard for the first level assessment of FXS and can detect the expanded allele even in mosaic fashion. This PCR based kit also allows the detection of AGG interruptions.

As the second-level analysis in the diagnostic flow-chart, CGG methylation testing can be performed to evaluate FMRP silencing. Dedicated kits, such as the Methylation-Sensitive Long-Range PCR (MS-LR-PCR) kit, have been developed to measure the methylation fraction of each FMR1 allele, using DNA after digestion with methylation-sensitive restriction enzymes [83]. This approach is also very useful to identify those rare FM unmethylated males that are asymptomatic carriers of a pathologic allele [83].

Finally, the finding of the loss of function mutations of FMR1 as causative of FXS prompted the development of specific molecular techniques. FMR1 sequence analysis and MLPA must therefore be offered to patients with a clinical phenotype highly suggestive of FXS but with a normal range of CGG repeats. Additional testing to identify intragenic deletions or duplications is required when PCR amplification fails, suggesting a possible exonic or whole-gene anomaly [6].

### Prenatal FMR1 testing

FXS molecular tests are usually performed postnatally on PBLs in the presence of the appropriate clinical criteria described in the clinical section. Moreover, it is also possible to perform a prenatal test of FXS using LR-PCR-based protocols on DNA from either chorionic villi or amniocytes. Currently, according to ACMG (American College of Medical Genetics) and ACOG (American Congress of Obstetricians and Gynaecologists) guidelines, FMR1 prenatal testing should be offered to couples with a personal or familial history of the following:

1. FXS- or FX-related disorders
2. Unexplained ID or developmental delay
3. Isolated cognitive impairment
4. Autism
5. Idiopathic familiar POI or elevated FSH at age <40 years
6. Isolated cerebellar ataxia with tremor

In addition, given the high incidence of FXS in the general population, a consistent number of genetic health professionals have supported prenatal testing for all women who request the analysis, regardless of their personal/familial history [84]. However, it should be taken into consideration that FMR1 test interpretation could be complicated by different problems; in particular, the presence of GZ alleles is very difficult to be interpreted and counselled because the risk of expansion, although very low, cannot be excluded [76]. The prenatal findings of FM females are also complex because the XCI pattern can modify the clinical phenotype. Moreover, the presence of post-zygotic mosaicism can complicate the diagnosis. This phenomenon can generate false results, especially in prenatal screening, because the clinical phenotype of the proband would not be available until birth.

Finally, it has been suggested that GZ and PM carriers with a positive family history of FX-associated disorders are at higher risk of expansion [65]. This is at least partly due to the presence and number of AGG interruptions in the parental allele. AGG trinucleotide repeats genotyping can be therefore offered to determine the number and location of AGG trinucleotide interruptions within the tract of CGG repeats of FMR1, particularly in female carriers of a GZ or a small PM allele [6, 65, 85].

Considering all of these issues, for couples who request FXS screening, the ideal test should be proposed in the preconception period: this could be advantageous because the couple will be allowed to make conscious reproductive decisions. In addition, the parents would also receive useful information for their personal health. Indeed, the identification of premutated females (which are at risk for POI) may allow a more effective reproductive intervention in those desiring a pregnancy.

Newborn screening for FXS has been proposed [86, 87], even if its application remains controversial. Although FXS is a relatively frequent disorder, and there would be the possibility to apply a quite sensitive and specific test, a real benefit of testing is lacking because there is no specific therapy currently to treat children, and treatment is essentially symptom based. In 2014, Godler et al. proposed a screening protocol where FMR1 methylation analysis would be used as the first-line test to selectively identify only FM carriers, followed by a triple-primed CGG-based test to confirm the positive results [87]. Nonetheless, to date, newborn screening for FXS is not performed in any country. The quest for a blood-based biomarker of FXS is currently underway [74]. Finding a simple and low-cost biochemical test would pave the way to include an FXS test in routine newborn screening.

### Patient management: therapeutic strategies and social issues

FXS emerges as a complex disease with a primary neuropsychiatric involvement but potentially affects more than one apparatus, therefore needing a large-scale intervention able to address all the physical, psychological and social implications of the disease. Treatment plans should be individualized based on the symptoms and age-related comorbidities of each individual. Speech and language therapy must be recommended to children, especially in that with early diagnosis. Most males show in fact moderate to severe delay in communication skills, while the communication skills of females are considerably less affected [48]. Behavioural therapy is another useful tool that helps normalize some of the symptoms, and it has been reported to be an effective approach for aggression in 71% of patients [37]. Therapy techniques that have been validated for autism are often helpful for FXS but must be modified based on the particular ASD features in FXS phenotype [88]. Physical therapy is needed in some cases, and occupational therapy must be offered to adults. A study by Martin et al., collecting males and females with FXS aged 0–63, showed that 72% of males and 47% of females receive one or more non-pharmacological therapy service [89]. The most common service for both males and females is speech–language therapy (68% males, 42% females), followed by occupational therapy (59% males, 30% females), physical therapy (25% males, 10% females), and behaviour management therapy (21% males, 6% females). Overall, males are more likely to receive therapy services as well as a greater number of services than females. All these strategies are mostly adopted during childhood and an age-related decline of therapies occurs for both males and females, with the use of services peaking by 11 years and essentially non-existent after the age of 20 [89].

Pharmacologic therapy may be recommended to improve behaviour problems (such as aggression, anxiety, hyperactivity, problems with impulse control, and poor attention span) and also to treat more severe disorders, such as ADHD or depression [8, 52]. The most common classes of medication used by FXS patients are selective serotonin reuptake inhibitors (SSRIs) and atypical anti-psychotics, with SSRIs use being more common among females than males [90]. In approximately 60% of patients, drug treatment for psychological issues helped the remission of some symptoms; in particular, stimulants can be used to improve attention and hyperactivity, SSRIs for anxiety, alpha-agonists for hyperactivity and overarousal, and antipsychotics for irritable and aggressive behaviours [91]. Drugs should anyway always be combined with non-pharmacological strategy to reach the best outcome. For ADHD in particular, it has been demonstrated that the early diagnosis and treatment play a role in improving both concurrent and long-term social functioning [33].

Lithium has been proposed as possible treatment, and in 2008, a pilot add-on trial has been conducted to evaluate the safety and efficacy of this drug in FXS patients. The results showed a significant improvement in hyperactivity, inappropriate speech, aggression, abnormal vocalizations, self-abuse, work refusal, outbursts, over-emotionality, anxiety, mood swings, tantrums, perseveration, crying, and maladaptive behaviour after 2 months of treatment [92]. Scores on the Clinical Global Improvement Scale were also significantly enhanced, and positive responses were distributed across all the age ranges of the study cohort, suggesting that both children and young adults with FXS can benefit from lithium treatment [92].

New targeted treatments for FXS (mGluR5 antagonists, GABA A and B agonists, minocycline) are now being studied. In Fmr1 knockout (KO) mice, the glutamatergic receptors signalling and/or localization is enhanced. New studies that try to reduce excitatory neurotransmission by antagonism of group I metabotropic glutamate receptors (mGluRs), particularly mGluR5, are still underway [93]. By now, studies conducted on Fmr1 KO mice demonstrated that the reduction of mGluR5 levels can normalize protein synthesis, dendritic spines, and some behaviour [94]. In FXS, an insufficient inhibitory GABAergic function has also been reported. A positive modulation of GABA(A) receptors has been demonstrated to improve some behavioural and neurophysiological alterations in Fmr1 KO mice [95]. Drugs modulating GABA signalling could therefore be an effective therapy. Minocycline is an FDA-approved treatment for acne and is known to have inhibitory effects on matrix metalloproteinase-9 activity. It is currently under study for FXS treatment and seems to have its greatest effect in young children, where it strengthens synaptic connections and enhances cognitive development [96].

Moreover, specific pharmacological intervention is needed to address common complications, such as epilepsy, metabolic disorders and hypertension.

Going beyond treatment, an important issue to consider in the management of FXS patients is the psychological health of their parents and caregivers; both have to take care of these complicated patients in different ways, and both have been proven to suffer psychological consequences.

Although a diagnosis of FXS is beneficial to the family for establishing the reason why a child has cognitive deficits and/or behavioural problems, all the family members have to cope with the disease and the derived stress. Mental and physical health problems especially affect families with higher stress levels, and these families often have an impaired ability to manage the difficult behaviours and physical problems of their children. This leads to an increased need for intervention with caregivers at the family level [37]. On the other hand, caregivers of patients with FXS are more likely to develop comorbidities such as anxiety, depression, stress, and sleep disorders than the caregivers of patients without FXS and are also more likely to receive medications for these conditions [97, 98].

Despite the relatively low incidence of FXS, this disease has a significant impact on parents, caregivers and entire society within which the patient lives. The poor social functioning of FXS individuals, together with the need for medication and non-pharmacological intervention, has important consequences in terms of the costs and resources employed. The percentage of working FXS patients varies largely with studies, in the range of 20–70%, with females being more frequently employed than males [97, 98]. Predictably, the higher functioning employed group of FXS patients also has lower direct healthcare costs [98]. It has been estimated that the economic burden of FXS in Europe is significant, with a mean annual cost per patient reaching up to €58,862 [97]. The patient cost is even higher in the USA, where it has been estimated to be as high as $14,677 every month [98].

## Conclusions

FXS is a well-known condition; however, most of the studies thus far have focused on its neuropsychiatric features. This review was aimed to provide an extensive overview of both the clinical and molecular features of this syndrome. Unfortunately, some of the studies cited in this work have limitations, such as the paucity of patients enrolled and the bias due to the collection of data in a single-country population, which may not be representative of the average global FXS population. Most of the studies collected focus on FXS children; however, in recent years, insight into the adult presentation of the disease has progressively increased. Furthermore, only a few studies have been conducted including non-Caucasian populations. Pharmacological treatment in FXS is essentially symptom based, but the growing understanding of the molecular and biological mechanisms of the disease are paving the way to targeted therapy, which might reverse the effects of FMRP deficiency and be a real cure for the disease itself, not just its symptoms.

## Abbreviations

ACMG: American College of Medical Genetics
ACOG: American Congress of Obstetricians and Gynecologists
ADHD: Anxiety disorder/hyperactivity disorder
ASD: Autism spectrum disorder
CTDs: Connective tissue disorders
FM: Full mutation
FXS: Fragile X syndrome
FXTAS: Fragile X-associated tremor/ataxia syndrome
GZ: Grey-zone
ID: Intellectual disability
IQ: Intellective quotient
mGluRs: Metabotropic glutamate receptors
MRI: Magnetic resonance imaging
MS-LR-PCR: Methylation-sensitive long-range PCR
PBLs: Peripheral blood lymphocytes
PM: Permutation
POI: Primary ovarian failure
SSRIs: Selective serotonin reuptake inhibitors
XCI: X chromosome inactivation
